# Heterogenous Clinical Landscape in a Consanguineous Malonic Aciduria Family

**DOI:** 10.3390/ijms222312633

**Published:** 2021-11-23

**Authors:** Sarah Snanoudj, Stéphanie Torre, Bénédicte Sudrié-Arnaud, Lenaig Abily-Donval, Alice Goldenberg, Gajja S. Salomons, Stéphane Marret, Soumeya Bekri, Abdellah Tebani

**Affiliations:** 1 Department of Metabolic Biochemistry, UNIROUEN, INSERM U1245, CHU Rouen, Normandie University, 76000 Rouen, France; sarah.snanoudj@chu-rouen.fr (S.S.); b.sudrie-arnaud@chu-rouen.fr (B.S.-A.); soumeya.bekri@chu-rouen.fr (S.B.); 2 Department of Neonatal Pediatrics, Intensive Care and Neuropediatrics, UNIROUEN, INSERM U1245, CHU Rouen, Normandie University, 76000 Rouen, France; stephanie.torre@chu-rouen.fr (S.T.); lenaig.donval@gmail.com (L.A.-D.); stephane.marret@chu-rouen.fr (S.M.); 3 Department of Genetics and Reference Center for Developmental Disorders, UNIROUEN, INSERM U1245, CHU Rouen, Normandie University, 76000 Rouen, France; alice.goldenberg@chu-rouen.fr; 4 Metabolic Unit, Department of Clinical Chemistry, Laboratory Genetic Metabolic Diseases, Department of Paediatric Metabolic Diseases, Emma Children’s Hospital, Amsterdam Neuroscience, Vrije Universiteit Amsterdam, 1081 HV Amsterdam, The Netherlands; g.salomons@amsterdamumc.nl; 5 Amsterdam Gastroenterology Endocrinology Metabolism, Vrije Universiteit Amsterdam, 1105 AZ Amsterdam, The Netherlands

**Keywords:** malonic aciduria, *MLYCD*, inborn errors of metabolism, beta-oxidation, cardiomyopathy

## Abstract

Malonic aciduria is an extremely rare inborn error of metabolism due to malonyl-CoA decarboxylase deficiency. This enzyme is encoded by the *MLYCD* (Malonyl-CoA Decarboxylase) gene, and the disease has an autosomal recessive inheritance. Malonic aciduria is characterized by systemic clinical involvement, including neurologic and digestive symptoms, metabolic acidosis, hypoglycemia, failure to thrive, seizures, developmental delay, and cardiomyopathy. We describe here two index cases belonging to the same family that, despite an identical genotype, present very different clinical pictures. The first case is a boy with neonatal metabolic symptoms, abnormal brain MRI, and dilated cardiomyopathy. The second case, the cousin of the first patient in a consanguineous family, showed later symptoms, mainly with developmental delay. Both patients showed high levels of malonylcarnitine on acylcarnitine profiles and malonic acid on urinary organic acid chromatographies. The same homozygous pathogenic variant was identified, c.346C > T; p. (Gln116*). We also provide a comprehensive literature review of reported cases. A review of the literature yielded 52 cases described since 1984. The most common signs were developmental delay and cardiomyopathy. Increased levels of malonic acid and malonylcarnitine were constant. Presentations ranged from neonatal death to patients surviving past adolescence. These two cases and reported patients in the literature highlight the inter- and intrafamilial variability of malonic aciduria.

## 1. Introduction

Malonic aciduria is an extremely rare inborn error of metabolism due to malonyl-CoA decarboxylase (MCD—EC. 4.1.1.9) deficiency (MIM 248360) with an autosomal recessive inheritance.

MCD, encoded by the *MLYCD* gene (MIM 606761), catalyzes the decarboxylation of malonyl-CoA to acetyl-CoA. *MLYCD* is located on the long arm of chromosome 16 (16q23.3) and contains five exons. Only one transcript is described (NM_012213). Two isoforms are produced using an alternative initiation codon. The longest isoform with 493 amino acids, chosen as the canonical sequence, is present in the mitochondria. The other isoform (454 amino acids) is found in the cytoplasmic and peroxisomal compartments [1]. In the mouse, the acetylation at p.Lys471 activates the enzyme [2], which does not require any cofactors or divalent metal ions [3]. Two main protein domains are described: an alpha-helical domain and a catalytic domain with two active sites. The formation of interchain disulfide bonds leads to positive cooperativity between active sites and increases the affinity for malonyl-CoA and the catalytic efficiency in vitro [4]. The protein forms homotetramers made of a dimer of dimers [4,5]. MCD is strongly expressed in the heart, liver, skeletal muscle, kidney, and pancreas. In the brain, placenta, spleen, thymus, testis, ovary, and small intestine, the expression is weaker [6,7].

**Figure 1:**
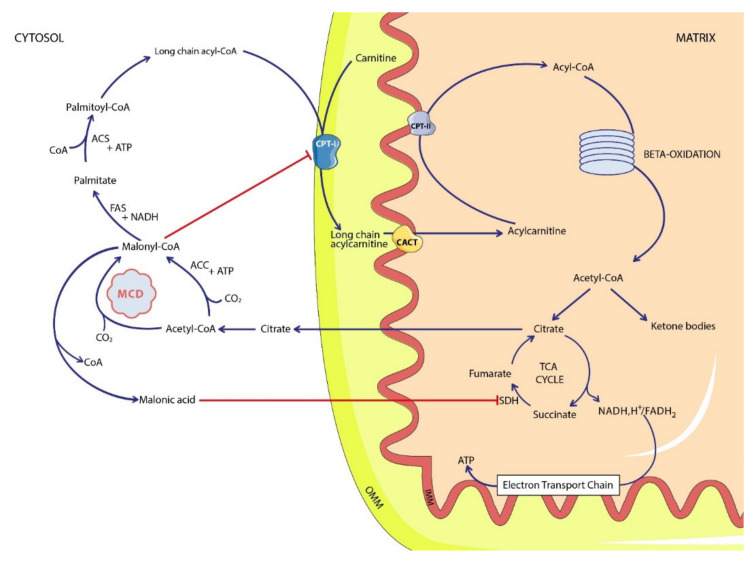
Simplified malonic acid pathway. *ACC*: acetyl-CoA carboxylase; *ACS*: acetyl-CoA synthetase; *CACT*: carnitine-acylcarnitine translocase; *CoA*: coenzyme A; *CPT*: carnitine-palmitoyl transferase; *FAS*: fatty acid synthase; *MCD*: malonyl-CoA decarboxylase; *SDH*: succinate dehydrogenase; *TCA cycle*: tricarboxylic acid cycle; *IMM*: inner mitochondrial membrane; *OMM*: outer mitochondrial membrane.

Malonyl-CoA is formed by carboxylating acetyl-CoA. This reaction is catalyzed by the acetyl-CoA carboxylase enzyme. Malonyl-CoA provides 2-carbon units to fatty acids and commits them to fatty acid chain synthesis. Long-chain fatty acids are then used as energy substrates through mitochondrial beta-oxidation. Free fatty acids are activated and form long-chain acyl-CoAs. They are transported into the mitochondria through the carnitine shuttle: acyl-CoA molecules are conjugated to carnitine by carnitine palmitoyl-transferase I (CPT-I). This is the limiting step of beta-oxidation. Acylcarnitines are transported across the inner mitochondrial membrane by the carnitine-acylcarnitine translocase. Acylcarnitines are converted into acyl-CoAs and released into the mitochondrial matrix via carnitine-palmitoyl transferase II (CPT-II). Subsequently, acyl-CoAs undergo beta-oxidation with the release of one molecule of acetyl-CoA at each cycle of oxidation [8]. Acetyl-CoA is used to produce ketone bodies or to fuel the Krebs cycle (or tricarboxylic acid cycle), which yields NADH and FADH_2_, providing the respiratory chain substrates for the production of ATP. Malonyl-CoA regulates the limiting step in beta-oxidation by inhibiting CPT-I, playing an important role in the regulation of mitochondrial beta-oxidation of fatty acids in fed conditions. Increased malonyl-CoA and malonic acid due to MCD deficiency result in the inhibition of the carnitine palmitoyltransferase I (CPT-I) and of the Krebs cycle (Figure 1).

In peroxisomes, MCD may be involved in degrading intraperoxisomal malonyl-CoA, which is generated by the peroxisomal beta-oxidation of odd chain-length dicarboxylic fatty acids. MCD plays a role in the metabolic balance between glucose and lipid oxidation in the muscle, independent of alterations in insulin signaling. It also plays a role in controlling the extent of ischemic injury by promoting glucose oxidation [3,4,6,7].

The MCD was first described in 1953 by Hayaishi [9], and MCD deficiency (MCDD or MLYCDD) was first reported by Brown et al. in 1984 [10]. Malonic aciduria is characterized by systemic clinical involvement, including neurologic and digestive symptoms, metabolic acidosis, hypoglycemia, failure to thrive, seizures, developmental delay, and cardiomyopathy, as malonic acid is most commonly present in the heart muscle.

Diagnosis of malonic aciduria is based on elevated levels of malonic acid in urine and of malonylcarnitine (C3DC) in plasma or whole blood. The molecular analysis of *MLYCD* confirms the diagnosis and allows for genetic counseling and prenatal diagnosis.

Treatment consists of a high-carbohydrate and low-fat diet. Carnitine supplementation and symptomatic treatment of cardiomyopathy are often needed. Early diagnosis can help prevent and treat cardiomyopathy and improve neurological development.

The present report describes two index cases belonging to the same family that, despite an identical genotype, present very different clinical pictures. We also provide a comprehensive literature review of reported cases.

## 2. Patients and Methods

### 2.1. Clinical Presentations

#### 2.1.1. Case 1

The first case is a two-year-old boy, born at term after a cesarean section for abnormal fetal heart rate. He was the second child of a consanguineous couple. The older sister is healthy. At 40 h of life, he exhibited abnormal movements, general hypertonia, clonies of the four limbs, with gray complexion and perioral cyanosis. Electroencephalogram (EEG) showed multiple seizures starting mostly in the right frontal lobe and in the right rolandic region that receded after a loading dose of phenobarbital. The EEG remained very poor and discontinuous with persistent axial hypotonia. Phenobarbital was stopped, and the use of sodium valproate and then levetiracetam normalized the EEG. Brain MRI performed at three days of life showed diffuse abnormalities in the white matter and basal ganglia. Dilated cardiomyopathy was found on cardiac echography.

#### 2.1.2. Case 2

The second case is a 5-year-old boy born prematurely at 28 weeks of gestation with low birth weight. His personal history was marked by sequelae of prematurity (bronchopulmonary dysplasia and spastic diplegia) and infantile asthma. The child presented with a developmental delay—he walked at 27 months, a language delay, and cleanliness was acquired at 5 years old. He also exhibited gastrointestinal dysfunction with vomiting, behavioral abnormalities with auto and hetero-aggressivity, and mild facial dysmorphia. Genetic testing for the most frequent causes of neurodisability (karyotype, fragile X syndrome, CGH-array) were negative. Basic biochemical workup was unremarkable.

### 2.2. Metabolic Investigations

#### 2.2.1. Acylcarninite Profile

Acylcarnitine profiles were obtained using an LC-MS/MS method (ChromSystems kit, Munich, Germany). Butylated esters of acylcarnitines are acquired by precursor ion scanning of 85 *m*/*z* in positive ion mode. Derivatized samples were injected into a 4000 QTRAP mass spectrometer (SCIEX, Framingham, MA, USA) by an autosampler. Isotopically labeled internal standards were used [11].

#### 2.2.2. Urinary Organic Acids

Urinary organic acid profiles were obtained using gas chromatography-mass spectrometry. The sample was subjected to derivatization with N,O-bistrimethylsilyl trifluoacetamide and trimethylchlorosilane. Derivatized samples were injected into a QP-2010 Plus GS-MS operation (Shimadzu, Kyoto, Japan) in split mode. The metabolites were analyzed as trimethylsilyl compounds. Heptadecanoic acid was used as an internal standard [11].

### 2.3. Molecular Analysis

This analysis was conducted in the Department of Clinical Chemistry, Metabolic Unit, VU University Medical Center, Amsterdam, The Netherlands (Pr. G. S. Salomons). Exonic regions, including intron/exon junctions and the promoter of *MLYCD*, were sequenced by conventional Sanger sequencing techniques, as previously described [12], using a BigDye™ Terminator v3.1 Cycle Sequencing Kit (ThermoFisher Scientific, Waltham, MA, USA) and an ABI 3100 Genetic Analyzer (Applied Biosystems, Waltham, MA, USA). The data were analyzed using Mutation Surveyor software (Softgenetics, State College, PA, USA). Standard nomenclature, according to Den Dunnen (http://www.hgvs.org/mutnomen/ accessed on 15 October 2021) was used to describe variants. The GenBank accession number (AF090834) was used as a reference for *MLYCD*.

### 2.4. Literature Review

A literature review was performed using the terms malonyl-CoA decarboxylase deficiency, malonic aciduria and *MLYCD*. Articles concerning suspected [13] or confirmed combined malonic and methylmalonic aciduria, and malonic aciduria in dogs, were excluded.

## 3. Results

### 3.1. Metabolic Investigations

#### 3.1.1. Case 1

Metabolic investigations at five days of life showed an abnormal acylcarnitine profile with high levels of malonylcarnitine (C3DC). Total and free carnitine were decreased. Urinary organic acid analysis allowed the identification of high malonic acid excretion and moderate increase of methylmalonic and ethylmalonic acids.

#### 3.1.2. Case 2

Urinary organic acids chromatography performed at five years of age revealed a major increase of malonic acid and a mild increase of methylmalonic acid. C3DC concentration was elevated.

**Figure 2:**
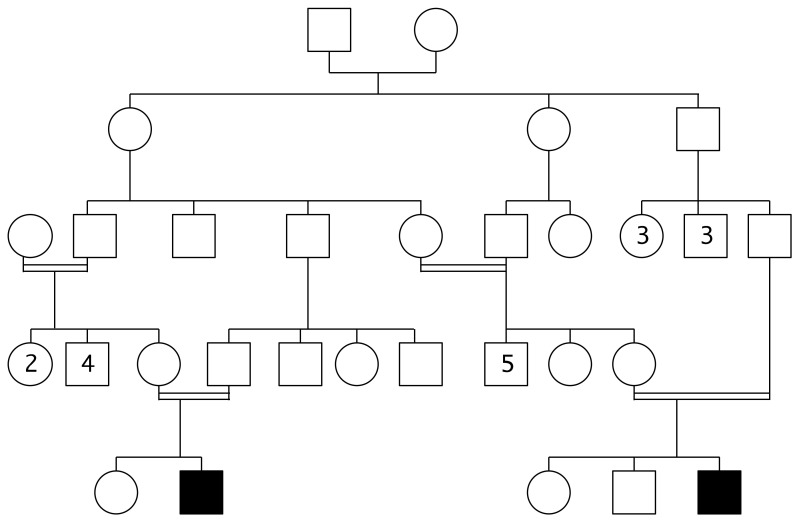
Family tree of the two cases represented as solid squares. Numbers in squares and circles refer to the number of siblings.

The diagnosis of this second case was made two years after the first one. The two families were initially not linked. Subsequently, genetic counseling and the family tree made it possible to connect the two cases (Figure 2).

### 3.2. Molecular Analysis

A subsequent molecular analysis allowed the identification of a homozygous variant NM_012213.2 (*MLYCD*): c.346C > T resulting in a premature stop codon (p. (Gln116*)) in both cases. The parents were heterozygous for this mutation. This nonsense variant had never been reported in the literature and was classified as pathogenic according to the American College of Medical Genetics (ACMG) recommendations [14]. The described variant has been added to Leiden Open Variation Database (LOVD).

### 3.3. Patients Follow-Up

#### 3.3.1. Case 1

Following the diagnosis of malonic aciduria, the boy was put on a diet restricted in long-chain fatty acids and rich in carbohydrate and medium-chain triglycerides. Carnitine supplementation (levocarnitine, 50 mg/kg/day) was introduced. The dilated cardiomyopathy was treated with furosemide and captopril.

His psychomotor development was delayed with persistent axial hypotonia and speech delay.

At 11 months old, he had an episode of hypoglycemia during an intercurrent infection. His weight and stature growth were within one standard deviation (SD); however, his bone age was 18 months old at 13 months old. Enteral feeding was started at 13 months of age because of gastrointestinal symptoms and feeding difficulties, and a gastrostomy was put in place at 16 months old.

#### 3.3.2. Case 2

The second case had, at follow-up, a normal cardiac function and no dysmorphia; he did not present clonies nor gastrointestinal disorders. He did, however, have microcephaly (−2SD) and a low weight (−1SD) for his age. He had a severe developmental delay and behavioral abnormalities with auto and hetero-aggressivity. He was treated with the same low-fat diet and carnitine.

### 3.4. Literature Review

**Figure 3:**
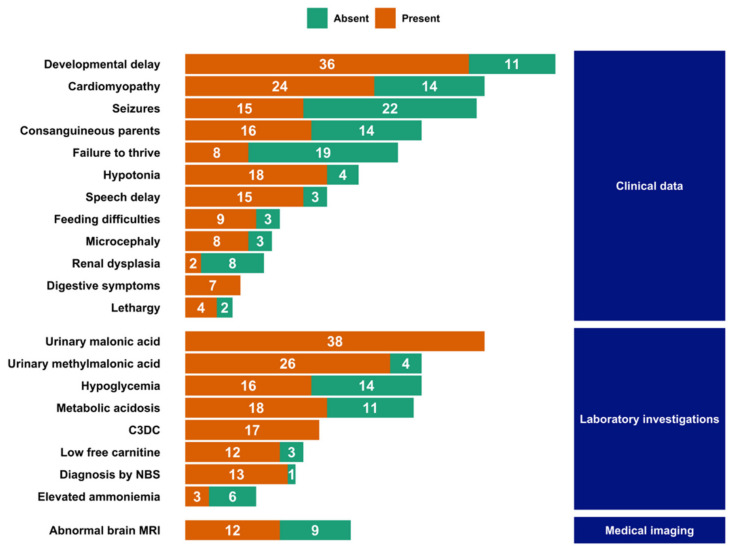
Phenotype summary of reported malonic aciduria cases in the literature. Orange bars indicated the presence of the sign, green bars its absence. *NBS*: newborn screening; *C3DC*: malonylcarnitine.

To the best of our knowledge, 52 patients have been described in the literature so far, in 26 articles or abstracts [10,12,15,16,17,18,19,20,21,22,23,24,25,26,27,28,29,30,31,32,33,34,35,36,37,38]. The most common symptoms described in those patients are developmental delay (77%, *n* = 36 out of 47 patients) and cardiomyopathy (63%, *n* = 24 out of 38) (Supplementary Table S1, Figure 3).

The cardiomyopathy is most often present at the time of the diagnosis, with a mean ejection fraction of 34%. Both dilated and hypertrophic myopathies are described, with the left ventricle being the most affected.

Other signs are common including hypotonia (82%, *n* = 18 out 22), speech delay (83%, *n* = 15 out of 18), seizures (41%, *n* = 15 out of 37), feeding difficulties (75%, *n* = 9 out of 12), and digestive symptoms (100%, *n* = 7).

In all patients, increased urinary excretion of malonic acid (with a mean value of 740 mmol/mol of creatinine) and C3DC were reported (in 38 patients out of 38 and 17 out of 17, respectively). Urinary methylmalonic acid excretion was also common (found in 26 patients out of 30—87% of patients), as well as low free carnitine (in 12 patients out of 15, or 80%). Acute manifestations included metabolic acidosis (in 18 patients out of 29, or 62%) and hypoglycemic episodes (16 out of 30, or 53%). Newborn screening (NBS) was performed in 14 cases, and the diagnosis was made in 13 of them [12,21,23,24,29,30]. Outside of patients diagnosed through NBS, diagnostic delays ranged from a few days to more than 10 years. The average delay was 17 months (over 22 cases, Supplementary Table S1).

Age at first symptoms varied. The most common initial presentations were a neonatal presentation with feeding difficulties, failure to thrive, somnolence with or without cardiac involvement, and a later presentation during childhood with developmental delay.

A brain MRI was performed in about one-third of the cases, and abnormalities were found in 57% (12 patients out of 21). The anomalies appear non-specific with most commonly generalized by atrophy, white matter diffuse hypersignal, and delayed myelination. Polymicrogyria was described in two cases. Sequelae of hypoglycemia could also be observed.

There was no difference between male and female patients in age at onset and at diagnosis, and in urinary malonic acid levels.

Seven pairs of siblings have been reported in the literature [12,16,20,24,29,34]. In two families, the diagnosis of one sibling allowed for a presymptomatic diagnosis in a younger sibling [13,22]. Conversely, in three families, an older sibling was diagnosed following acute presentation and ensuing diagnosis of a younger sibling [12,34].

**Figure 4:**
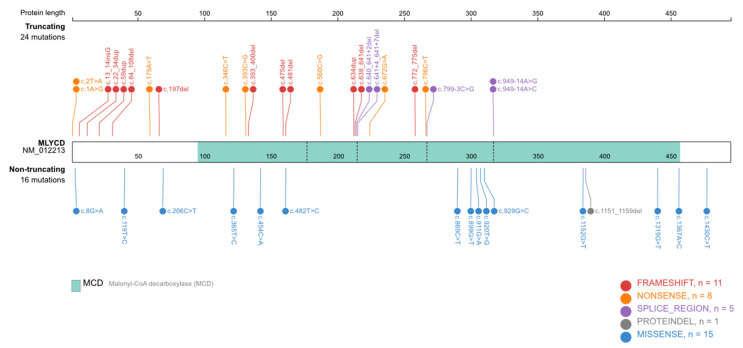
Molecular variations described in the literature represented along the MLYCD protein. Limits between the five exons are represented by the dotted lines. In blue, the decarboxylase protein domain. Twenty-four variations are truncating variants, including nonsense, frameshift indels, and splicing variants, and 16 are missense variants or deletion without frameshift. Nonsense variants include the two losses of start codon variants. The full list is presented in Supplementary Table S2. Large deletions are not reported on this figure. The figure was generated using https://proteinpaint.stjude.org/ (accessed on 15 October 2021).

Forty-six unique variants have been reported in the literature. The variant reported here has never been described, bringing the total to 47 *MLYCD* variants. Different types of variants are observed, 7 large deletions, including a complete gene deletion, 15 missense, 6 nonsense, 11 small insertions/deletions (indels) with a frameshift, 1 without, 2 variants concerning the first codon, and 5 intronic variants, with 2 different substitutions at the same position (c.949-14A). Forty-eight% (*n* = 19) of punctual variants are located in the first exon (Figure 4). Except for large deletions (deletion of the first 3 exons, deletion of all 5 exons, and exon 5 deletion) that have been described in two distinct index cases, all variants are private mutations.

Sixteen families out of 30 (53%) were consanguineous. In one family, no variant was found (*MLYCD* sequence was normal), in two families, only one variant could be identified at the heterozygous state, and in four families, no molecular diagnosis was reported.

## 4. Discussion

Malonic aciduria is a rare inborn error of metabolism. The phenotypic spectrum is large, but cardinal signs include developmental delay, cardiomyopathy, and metabolic manifestations with hypoglycemia and metabolic acidosis. Here, we describe two cousins from a consanguineous family with malonic aciduria. Although they harbored the same homozygous mutation, the severity of the disease was different with neonatal-onset, acute metabolic symptoms, and cardiomyopathy in one cousin, and a later onset with no acute metabolic manifestations in the other one.

We provide an extensive review of published cases, underlying the variability in severity of MLYCD deficiency within the clinical spectrum. The two patients described here are representative of the patients reported in the literature, with typical presentations, albeit at different ends of the spectrum, and a private variant. It highlights the varying degrees of severity that can be observed, ranging from neonatal death to patients surviving past adolescence. A genomic analysis could unveil the presence of modifier genes and help to understand the phenotypic variability within a particular genetic background.

Malonic acid is an inhibitor of multiple metabolic pathways (Figure 1) and has pleiotropic roles. This component has been detected in cytosolic, mitochondrial, and peroxisomal compartments, which may underlie the systemic clinic features. Regarding the cardiomyopathy, mitochondrial function is required for embryological compaction of the left ventricular myocardium. Moreover, mitochondrial β-oxidation impairment leads to a cardiac energetic deficiency. It was speculated that the developmental delay associated with MCD deficiency might be linked to the inhibition of peroxisomal β-oxidation, which is required for polyunsaturated fatty acid (PUFA) endogenous synthesis [1]. PUFA are known to be critical for normal brain development.

The rationale behind metabolic anomalies is multifold. Hypoglycemia can be due to the inhibition of different pathways: of mitochondrial β-oxidation via the inhibition of CPT-I by malonyl-coA, of pyruvate carboxylase secondary to dicarboxylic aciduria, and of the tricarboxylic acid cycle via the inhibition of succinic acid dehydrogenase by malonic acid. Metabolic acidosis is the first cause of early death and is largely explained by MLYCDD pathophysiology. Phenotypic overlap with fatty acid oxidation disorders in which hypoglycemia, lactic acidosis, and cardiomyopathy are common findings may be explained by the roles of MCD in the fatty acid metabolic pathway.

The diagnosis of malonic aciduria is relatively easy, as elevations of malonyl-carnitine and malonic acid are found on usual metabolic investigations (i.e., acylcarnitine profile and chromatography of urinary organic acids, respectively). However, these analyses are not part of the basic biochemical workup and are not available in every hospital facility. This highlights the need for specific training of clinicians and the necessity of a metabolic workup in a specialized laboratory in case of patients with metabolic features or unusual organ damage for the age. Apart from improving care, this would greatly reduce diagnostic wandering, which can linger for several years. As we have seen here, the average diagnostic delay in the literature was 17 months.

Newborn screening is also a powerful tool to fast-track diagnoses. In recent years, malonic aciduria has been included in newborn screening programs of several countries [39]. However, some patients may have a low malonic acid excretion at birth, and the diagnosis can be missed [30]. It is, therefore, important not to rule out this diagnosis if signs appear later in life. Conversely, symptoms may be apparent in the first hours or days of life, rendering NBS less determinant. The decision to include this very rare disease into NBS programs is motivated by the availability of a treatment that can improve the course of the disease, particularly if started early [24]. The etiological treatment is essentially nutritional: a diet low in long-chain fatty acids and rich in carbohydrates and medium-chain triglycerides with carnitine supplementation. This diet improves cardiac function and induces lower malonic acid excretion, although levels of C3DC excretion do not reflect the clinical severity. It does not prevent the occurrence of cardiomyopathy but limits its severity [21,24].

The main differential diagnosis of malonic aciduria is combined malonic and methylmalonic aciduria (CMAMMA). *ACSF3* was identified as the involved gene in 2011 [40]. It is suggested that CMAMMA is a benign condition, although a minority of patients do exhibit symptoms similar to some found in MLYCDD [41], as well as neurological manifestations in older patients [42]. False-positive results with elevated C3-DC on MS/MS acylcarnitine screen have also been found through a newborn screening program due to contamination of the fresh blood on the filter paper by chemicals used to disinfect surfaces [43].

## 5. Conclusions

Although malonic aciduria is a very rare inborn error of metabolism with a large phenotypic spectrum, the combination of cardinal signs involving multiple organs can steer clinicians towards metabolic investigations and subsequent diagnosis. A timely diagnosis is all the more important until a treatment can be put in place to mitigate or avoid complications. With the introduction of MCD deficiency in newborn screening programs, the number of patients is expected to rise, allowing a better understanding of the pathophysiology, the natural history of the disease, and the inter- and intrafamilial variability.

## Data Availability

All the data that support the findings are presented in the manuscript and the Supplementary Materials.

## Supplementary Materials

The following are available online at https://www.mdpi.com/article/10.3390/ijms222312633/s1.
